# A novel prognostic signature based on N7-methylguanosine-related long non-coding RNAs in breast cancer

**DOI:** 10.3389/fgene.2022.1030275

**Published:** 2022-10-13

**Authors:** Zhidong Huang, Kaixin Lou, Hong Liu

**Affiliations:** The Second Surgical Department of Breast Cancer, Tianjin Medical University Cancer Institute and Hospital, National Clinical Research Center for Cancer, Key Laboratory of Cancer Prevention and Therapy, Tianjin's Clinical Research Center for Cancer, Tianjin, China

**Keywords:** breast cancer, m7G-related lncRNA, prognostic signature, tumor immune cell infiltration, tumor mutational burden

## Abstract

Long non-coding RNA (lncRNA) are closely associated with the occurrence and progression of tumors. However, research on N7-methylguanosine (m7G)-related lncRNA in breast cancer is lacking. Therefore, the present study explored the prognostic value, gene expression characteristics, and effects of m7G-related lncRNA on tumor immune cell infiltration and tumor mutational burden (TMB) in breast cancer. lncRNA expression matrices and clinical follow-up data of patients with breast cancer were obtained from The Cancer Genome Atlas, revealing eight significantly differentially expressed and prognostically relevant m7G-related lncRNAs in breast cancer tissues: *BAIAP2-DT*, *COL4A2-AS1*, *FARP1-AS1*, *RERE-AS1*, *NDUFA6-DT*, *TFAP2A-AS1*, *LINC00115*, and *MIR302CHG*. A breast cancer prognostic signature was created based on these m7G-related lncRNAs according to least absolute shrinkage and selection operator Cox regression. The prognostic signature combined with potential prognostic factors showed independent prognostic value, reliability, and specificity. Meanwhile, we constructed a risk score-based nomogram to assist clinical decision-making. Gene set enrichment analysis revealed that low- and high-risk group were associated with metabolism-related pathways. Our study demonstrated the association between tumor immune cell infiltration based on analyses with the CIBERSORT algorithm and prognostic signature. We also assessed the correlation between prognostic signature and TMB. Lastly, quantitative real-time polymerase chain reaction analysis was performed to validate differentially expressed lncRNAs. The effective prognostic signature based on m7G-related lncRNAs has the potential to predict the survival prognosis of patients with breast cancer. The eight m7G-related lncRNAs identified in this study might represent potential biomarkers and therapeutic targets of breast cancer.

## 1 Introduction

Breast cancer (BC) is the most common malignancy worldwide (Winters et al., 2017), and its mortality rate is increasing every year. Among all malignant diseases, representing approximately 23% of cancer-related deaths, BC is considered a leading cause of death in postmenopausal women (Akram et al., 2017). The World Health Organization emphasizes that early diagnosis remains the most critical approach for improving the outcomes and survival rate of patients with BC (Bray et al., 2018). Therefore, it is indispensable to explore novel prognostic biomarkers and develop further measures for the diagnosis and treatment of BC.

Multiple mechanisms intertwine to ensure the correct and timely expression of each gene, with several of these mechanisms targeting the life cycle of RNA molecules, from transcription to translation (Batista, 2017; Zhang et al., 2019a). The modification of RNA have been reportedly demonstrated a crucial link with the development of cancer, as well as cardiovascular, metabolic, neurological, and other diseases, because of their reversibility, dynamics, and involvement in important biological processes (Jonkhout et al., 2017). The rapid development of RNA methylation profiling technologies and high-throughput sequencing (Qiang et al., 2018; Zou et al., 2019; Hasan et al., 2020; Tang et al., 2021) has revealed that N7-methylguanosine (m7G) modification is a considerable portion of RNA modifications.

As one of the most prevalent RNA modifications, m7G modifications are usually located in the 5′ cap and inner position of eukaryotic mRNAs or within rRNA and tRNA (Zhang et al., 2019b; Song et al., 2020). To date, studies on m7G primary focused on methylases of m7G, including the Trm8p/Trm82p heterodimer complex in yeast and the corresponding homologous methyltransferase-like protein-1 (METTL1) and WD repeat domain 4 (WDR4) proteins in humans. It has been reported that the METTL1/WDR4 complex could stabilize the tertiary structure of tRNA through the installation of m7G modifications at site G46 of diverse tRNA variable loops (Shaheen et al., 2015). In addition, the METTL1/WDR4 complex promotes miRNA biogenesis by modifying primary miRNA transcripts with m7G (Pandolfini et al., 2019). In addition, research has confirmed that the m7G modification is tightly correlated to tumor development and progression. In intrahepatic cholangiocarcinoma, the methylase METTL1 mediates m7G tRNA modification, selectively regulating the translation of oncogenic transcripts, including genes involved with the cell cycle and epidermal growth factor receptor (EGFR) pathways (Chen Z. et al., 2021). In hepatocellular carcinoma, c-Myc (MYC) activates WDR4 transcription and facilitates the stability and translation of CCNB1 mRNA through m7G modification, affecting the phosphorylation of PI3K and AKT and promoting P53 ubiquitination, ultimately fueling the progression of hepatocellular carcinoma (Chen Z. et al., 2021). In BC, the proliferative activity of BC cells is approximately 35% higher in patients with *PIK3CA* mutations, which are dependent on the m7G regulator mRNA cap methyltransferase (RNMT). As such, RNMT-targeted therapies in patients with *PIK3CA* mutations have better developmental prospects (Dunn et al., 2019). However, further m7G RNA methylation studies are needed to explore the mechanisms underlying cancer development.

Advances in genome sequencing technology have revealed that most of the genome does not encode proteins; nevertheless, non-coding genetic material is of great importance to various biological processes, like DNA methylation and RNA modification. Non-coding RNAs can be divided into two major categories based on their length: short non-coding RNAs (e.g., miRNAs and snRNAs) and long non-coding RNAs (lncRNAs). lncRNAs, a large group of structurally complex RNA genes, could regulate gene expression by interacting with DNA, RNA, or protein molecules and play cellular roles through various mechanisms. lncRNAs have been proposed as biomarkers of cancer (Lai et al., 2020; Li et al., 2021). For instance, *CAT104*, *LINC01234*, and *STXBP5-AS1* have been confirmed to predict the prognosis of patients with BC (Fernandes et al., 2019). Compared with the healthy controls, plasma lncRNA *HULC* concentrations are higher in patients with hepatocellular carcinoma (Parasramka et al., 2016). Likewise, overexpressed in prostate cancer, *PCA3* is considerated as a diagnostic biomarker and therapeutic target, which is a prostate-specific lncRNA (Lee et al., 2011). Identifying the differential expression of lncRNAs in tumors, which play roles in promoting both tumorigenesis and tumor suppression, provides an opportunity to develop new cancer therapies based on targeting lncRNAs.

**FIGURE 1:**
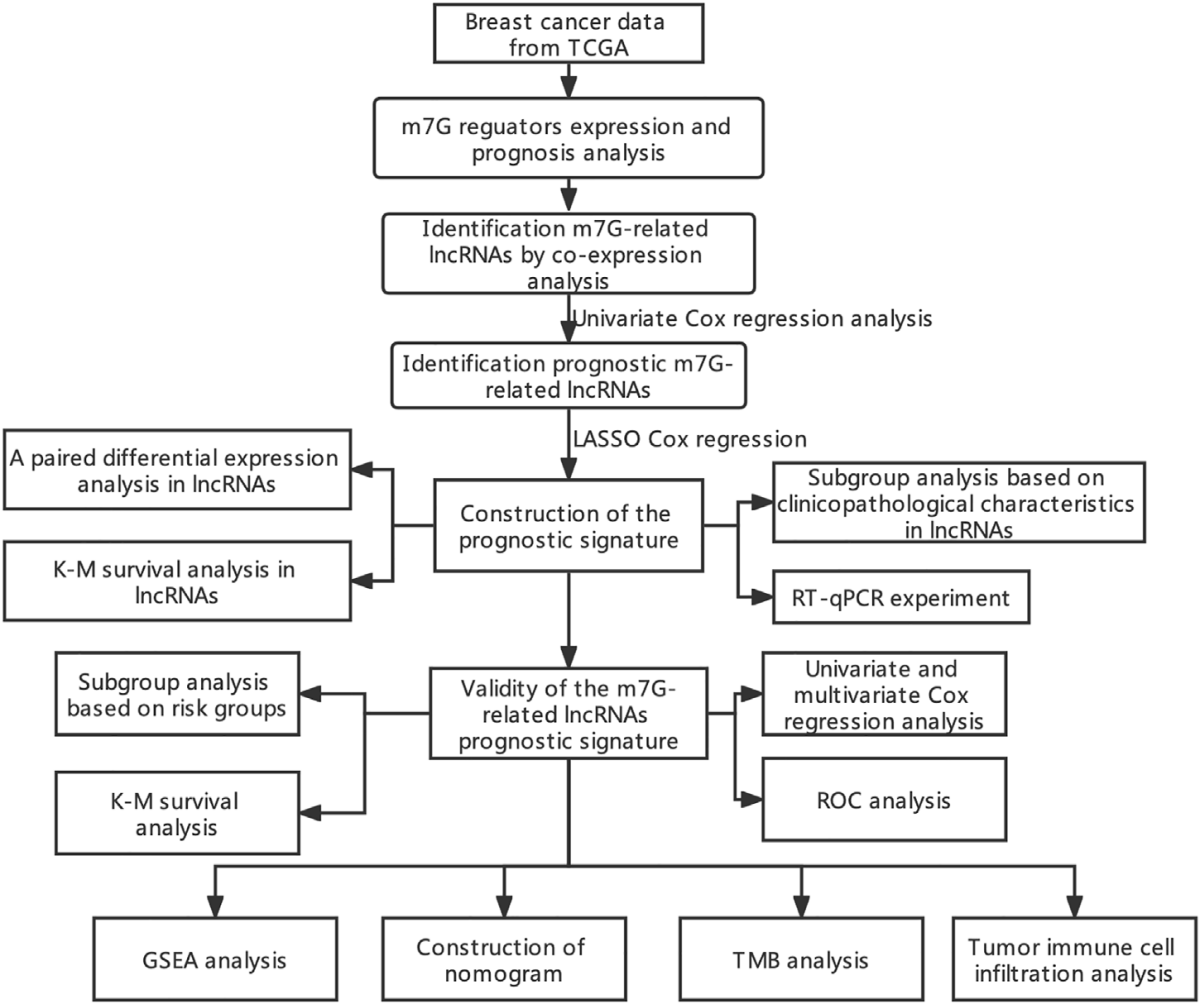
Flow chart of the study.

Currently, studies on the interaction between m7G modification and lncRNAs in BC are lacking. Thus, the purpose of present study is to explore the prognostic ability, gene expression features, clinical value, and predictive value on tumor immune cell infiltration and TMB of m7G-related lncRNAs in BC. To this end, we identified prognostic m7G-related lncRNAs in BC, created a prognostic risk signature, and further developed a nomogram according to the risk score, presenting a tool with promising prognostic value for BC (Figure 1).

## 2 Materials and methods

### 2.1 Data collection and processing

The lncRNA expression and clinical follow-up data shown in this research were collected from The Cancer Genome Atlas (TCGA) database (http://cancergenome.nih.gov/). Data from 1,222 breast tissues, including 1109 BC and 113 normal tissues, were analyzed. Table 1 presents the clinicopathological characteristics of the patients.

**TABLE 1:** Clinical characteristics of breast cancer patients in the training cohort.

Variables	No. of patients	Percentage (%)
Age (years)		
≤55	471	42.9
>55	626	57.1
Unknown	19	1.7
Gender		
Female	1,085	98.9
Male	12	1.1
Pathological stage		
I	183	16.7
II	621	56.6
III	249	22.7
IV	20	1.8
Unknown	24	2.2
T stage		
T1	281	25.6
T2	635	57.9
T3	138	12.6
T4	40	3.6
Unknown	3	0.3
N stage		
N0	516	47.0
N1	364	33.2
N2	120	10.9
N3	77	7.0
Unknown	20	1.8
M stage		
M0	912	83.1
M1	22	2.0
Unknown	163	14.9

### 2.2 Identification of m7G-related lncRNAs

From previously published studies, we extracted 40 m7G regulators; gene sets were selected from the GSEA (https://www.gsea-msigdb.org) database (“GOMF_M7G_5_PPPN_DIPHOSPHATASE_ACTIVITY”, “GOMF_RNA_7_METHYLGUANOSINE_CAP_BINDING”, and “GOMF_RNA_CAP_BINDING”). Pearson correlation analysis was performed using the “limma” R package to select m7G-lncRNA. The m7G-lncRNA pairs with a correlation coefficient >0.4 and *p* < 0.001 were kept. A total of 429 m7G-related lncRNAs were identified. The “dplyr,” “ggalluvial,” and “ggplot2” R packages were used to visualize the results of the m7G-lncRNA co-expression network as Sankey diagrams.

### 2.3 Selection of prognostic m7G-lncRNAs

First, we conducted a univariate Cox proportional hazards analysis with a *p* < 0.01 to select m7G-related lncRNAs that has association with the survival of patients with BC. Subsequently, using the least absolute shrinkage and selection operator (LASSO) Cox regression analysis, the best prognostically relevant lncRNAs were selected to build the prognostic signature. Gene interaction networks and Sankey plots were generated using Cytoscape 3.8, “dplyr,” “ggalluvial,” and “ggplot2” R packages.

### 2.4 Development and validation of the m7G-lncRNA prognostic signature (m7G-LPS) and nomogram

We used the “glmnet” R package to develop a lasso signature, which optimizes the L1 regularization parameter lambda through a built-in cross-validation function. With the help of the following formula, we calculated the risk score for each patient:

$$Risk\;score=\overset{n}{\underset{i=1}{\sum }}Coefi\;*\;xi$$

where *Coef*_*i*_ and *x*_*i*_ represent the survival-related regression coefficient and expression of each m7G-lncRNA, respectively.

Thereafter, based on the median of the prognostic risk score, the patient was assigned to either low- or high-risk groups. The heat map and scatter plots were generated using the heatmap function in R. The survival curves were plotted with the Kaplan-Meier method and adopted to analyze the discrepancy in overall survival (OS) between patients in the low- and high-risk groups. Univariate and multivariate Cox regression analyses were implemented to evaluate the independence of the risk score in predicting prognosis compared to other clinical variables. With the aid of the R package “ROCR,” the performance of the prognostic signature was evaluated by receiver operating characteristic (ROC) curve analysis. We then developed a nomogram using the R library “rms” package based on the independent prognostic factors for the clinical quantitative prediction of survival in patients with BC. Nomogram calibration was assessed using calibration plots. The genomes, which contains m7G genomes, m7G-lncRNA genomes, and m7G-LPS group expression profiles were implemented for effective dimensionality reduction, pattern recognition, and exploratory visual analysis through principal component analysis (PCA).

### 2.5 Gene set enrichment analysis (GSEA) analysis

GSEA analysis was performed to identify potential biological signaling pathways involved in low- and high-risk groups. When the |normalized enrichment score| > 1, nominal *p*-value < 0.05, and false-discovery rate q-value < 0.25, the pathways were defined as significantly enriched.

### 2.6 Correlation between the prognostic signature and tumor immune cell infiltration

The CIBERSORT with the LM22 gene set that we obtained from the CIBERSORT website was utilized to estimate the total immune infiltration in each BC sample and immune cell subsets (http://cibersort.stanford.edu/). Defining 22 human immune cell subtypes, LM22 is an annotated gene signature matrix containing 547 marker genes (e.g., dendritic cells, T cells, and B cells). With the aim of improving the accuracy of the deconvolution algorithm, 100 permutations of the default signature matrix to calculate the CIBERSORT *p*-values and root mean square errors for each sample file were implemented. Subsequently, regarding the differences in immune cell infiltration between the low- and high-risk groups, we utilized a threshold of *p* < 0.05 to analyze the differences by screening BC data. Spearman’s test was performed to assess correlations among different tumor immune cell types.

### 2.7 Analysis of tumor mutational burden

We obtained the somatic mutation data of BC from the TCGA database and calculated the tumor mutational burden (TMB) of each BC sample. We investigated the difference in TMB between the high-risk and low-risk groups and visualized it using “maftool,” “limma,” and “ggpubr” R packages. We obtained the optimal TMB cut-off value according to the algorithm in the “survminer” R package, and divided all samples into the high-TMB and low-TMB groups. We drew the Kaplan–Meier survival curve of high-TMB and low-TMB groups and analyzed the difference in the OS using the “survival” R package. The high- and low-TMB groups were further divided based on the prognostic signature into four groups: high-TMB and high-risk, low-TMB and high-risk, low-TMB and low-risk, and low-TMB and high-risk.

### 2.8 Cell culture

Breast cancer cell lines MCF7 were cultured in Dulbecco’s Modified Eagle Medium (DMEM, Gibco) supplemented with 10% FBS and 100 U/mL Penicillin/Streptomycin in a 5% CO2 incubator. Human normal breast cell lines MCF-10A were cultured in DMEM-F12 medium supplemented with 10% fetal bovine serum, 100 μg mL^−1^ epidermal growth factor (EGF), 1 mg mL^−1^ hydrocortisone, 10 mg mL^−1^ insulin, 100 U mL^−1^ penicillin G and 100 μg mL^−1^ streptomycin. Cells were collected at 90% confluence, and the medium was changed every 24–48 h.

### 2.9 RNA extraction and reverse transcription-quantitative polymerase chain reaction (RT-qPCR)

Gene expression for eight m7G-related lncRNA was measured by RT-qPCR. Total RNA was obtained from MCF-10A and MCF-7 cells using TRIzol reagent (TAKARA, Japan). The cDNA was synthesized with RNA Transcription Kit (TAKARA, Japan) and RT-qPCR was performed using SYBR Premix Ex Taq II (TAKARA, Japan). Expression was measured using CT values, normalized to that of GAPDH (ΔΔCT = ((CT (target, test) −CT (reference, test)) − (CT (target, calibrator) − CT (reference, calibrator)), and then expressed as 2-ΔΔCT. All RT-qPCR primers are listed in Table 2.

**TABLE 2:** Primer sequences used for RT-qPCR.

Primer	Sequence 5′ to 3′
BAIAP2-DT- F	CAT​CCA​GAG​ATC​GCC​CTG​AC
BAIAP2-DT- R	GTC​AGG​TTC​CAC​AGC​TAC​CC
COL4A2-AS1-F	TGT​GGG​ATG​GAG​ACA​TCC​TGA
COL4A2-AS1-R	CAG​AGC​TGT​TCC​AAA​ATG​CCA
FARP1-AS1-F	CAGGTGGATGGAAAGAGG
FARP1-AS1-R	AGATCACGGAGATGGTGG
RERE-AS1-F	CCC​AGG​AAG​GCA​GAC​AGA​TAA
RERE-AS1-R	CTC​GGG​GGA​GCT​GTA​GTT​TG
NDUFA6-DT-F	CTG​CCG​TCT​TAT​CCC​AGG​AG
NDUFA6-DT-R	GAG​ACG​TTC​AGT​CGA​AGC​CC
TFAP2A-AS1-F	ATT​GCT​CGC​CAG​TAC​CAC​AA
TFAP2A-AS1-R	GTG​GCG​GAA​TTG​GGG​TAA​GA
LINC00115-F	GCT​TTT​TGT​GGC​CAA​ACC​CA
LINC00115-R	CTC​AGT​GAC​GGA​ACC​GGA​C
MIR302CHG-F	TGT​TCC​TGC​TTG​TGG​TGC​AT
MIR302CHG-R	AAA​GTT​GAA​GGG​AGC​CCA​CC
GAPDH-F	GGT​GTG​AAC​CAT​GAG​AAG​TAT​GA
GAPDH-R	GAG​TCC​TTC​CAC​GAT​ACC​AAA​G

### 2.10 Statistical analysis

Kruskal–Wallis or Wilcoxon tests were used for intergroup comparisons of the differences in the expression of m7G regulators and m7G-related lncRNAs, the clinicopathological parameter, the proportion of the 21 tumor-infiltrating immune cell subtypes, and TMB in high- and low-risk groups. Two-sided log-rank tests were performed to compare Kaplan–Meier OS curves. All statistical analyses were carried out using software R (version 4.2.1). *p*-values < 0.05 were regarded as indicating statistically significant differences.

## 3 Results

### 3.1 Identification of m7G-related lncRNAs and construction of the prognostic signature

**FIGURE 2:**
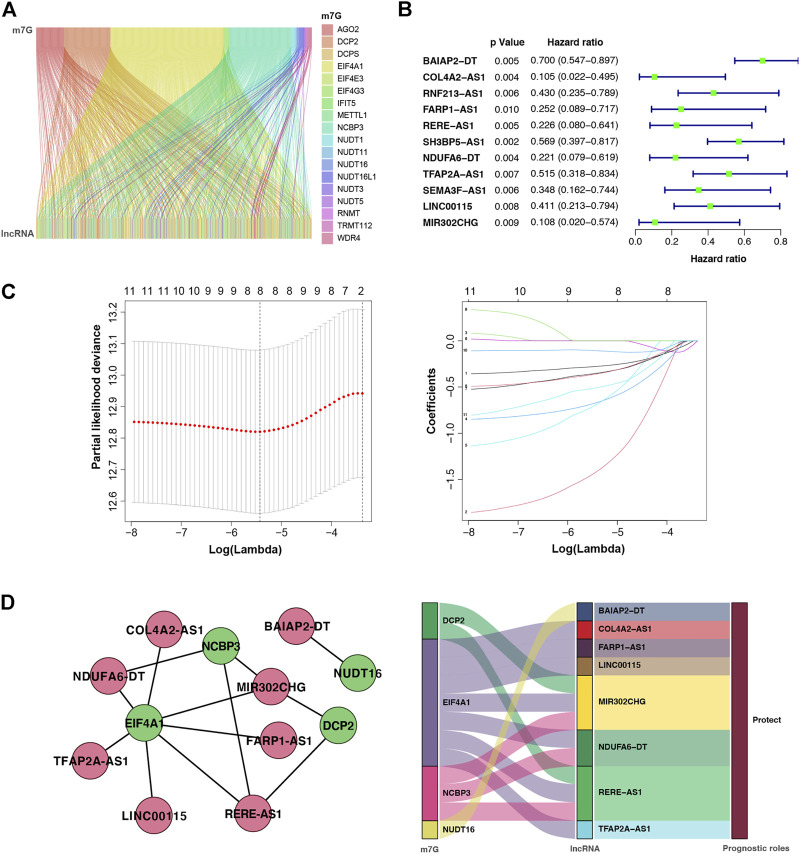
m7G-associated lncRNAs (m7G-lncRNAs) and their co-expression networks with significant prognostic value in breast cancer. **(A)** Sankey plot showing the relationship between m7G and m7G-lncRNAs. **(B)** Forest diagram of univariate Cox regression analysis of m7G-lncRNAs. **(C)** A LASSO Cox regression was used to select independent factors and construct a prognostic signature. **(D)** Network and Sankey plot showing the connection of m7G and eight m7G-lncRNAs with independent prognostic value.

To explore the role of m7G regulators in BC, we analysis the expression of m7G-related genes in breast cancer. Supplementary Figure S1 shown that 31 genes are differentially expressed in breast cancer. In addition, patients with different m7G-related gene expression levels have different prognosis in breast cancer, despite the lack of statistical significance which needs to be further improved in the future work (Supplementary Figure S2). But we can still see the significance of m7G in breast cancer. On the basis of the co-expression analysis in TCGA database, the lncRNAs of 387 genes were identified as co-expressed with m7G (Figure 2A). Further, the prognosis of BC was tightly associated with 11 m7G-related lncRNAs using univariate Cox regression analysis (*p* < 0.001): *BAIAP2-DT*, *COL4A2-AS1*, *RNF213-AS1*, *FARP1-AS1*, *RERE-AS1*, *SH3BP5-AS1*, *NDUFA6-DT*, *TFAP2A-AS1*, *SEMA3F-AS1*, *LINC00115*, and *MIR302CHG* (Figure 2B). Among them, eight m7G-related lncRNAs were further selected to construct a prognostic indicator based on the LASSO Cox regression algorithm, namely, *BAIAP2-DT*, *COL4A2-AS1*, *FARP1-AS1*, *RERE-AS1*, *NDUFA6-DT*, *TFAP2A-AS1*, *LINC00115*, and *MIR302CHG* (Figure 2C). The coefficients of the eight selected genes calculated by LASSO regression analysis are shown in Table 3. The m7G-associated lncRNA–mRNA interaction network consisted of four m7G regulators and eight lncRNAs, as shown in Figure 2D, demonstrating that the m7G regulator EIF4A1 is a key node co-expressed with seven lncRNAs and the prognostic role of all lncRNAs in BC are protective factors.

**TABLE 3:** m7G-lncRNAs selected to build m7G-LPS and the corresponding coefficients.

lncRNAs	Coefficients
BAIAP2-DT	−0.27758412746579
COL4A2-AS1	−1.41967228671049
FARP1-AS1	−0.671077622897499
RERE-AS1	−0.69129846535715
NDUFA6-DT	−0.352552610278439
TFAP2A-AS1	−0.360410426066037
LINC00115	−0.107203641246423
MIR302CHG	−0.508621483491941

### 3.2 Validation of the clinical significance of eight m7G-lncRNAs

**FIGURE 3:**
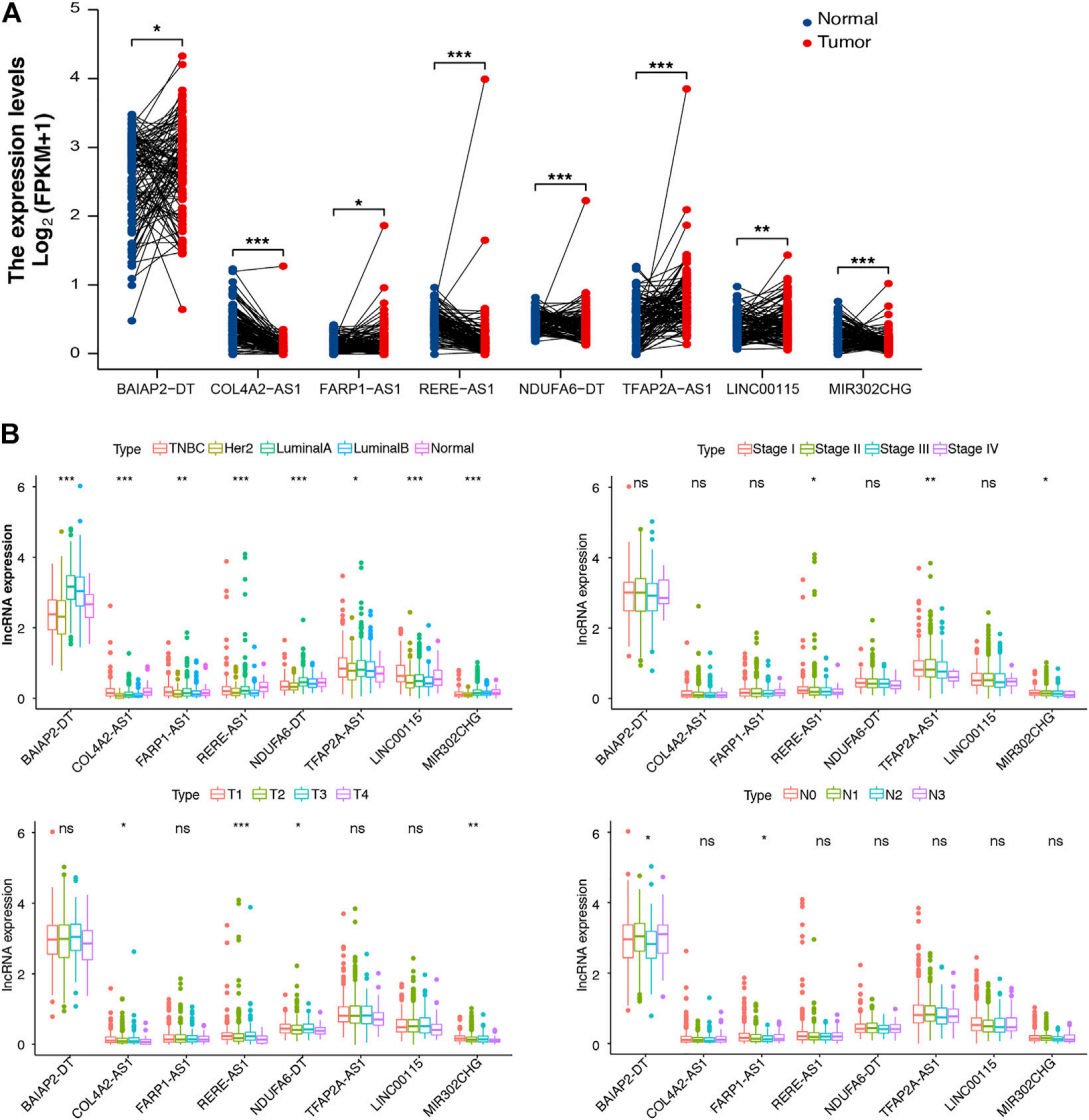
Differential expression analysis of m7G-associated lncRNAs (m7G-lncRNAs). **(A)** A paired differential expression analysis of the eight prognosis-related m7G-lncRNAs in normal and breast cancer (BC) tissues. **(B)** Differential expression analysis of m7G-lncRNAs in BC tissues according to molecular subtype, histological stage, T stage, and N stage (ns: not significant; *p < 0.05; * *p < 0.01; * * *p < 0.001).

To support the clinical significance of these lncRNAs, a paired differential expression analysis was performed, revealing significant group differences in all lncRNAs. Specifically, *COL4A2-AS1* and *MIR302CHG* expression was high in normal tissues (Figure 3A). In addition, we conducted the Kaplan–Meier survival analysis, Supplementary Figure S3 revealed that *BAIAP2-DT*, *COL4A2-AS1*, *RERE-AS1*, *NDUFA6-DT*, *TFAP2A-AS1*, and *LINC00115* were associated with good prognosis. Last, we examined the relationship between m7G-lncRNA expression and clinicopathological characteristics, demonstrating significant differences between different molecular subtypes of BC (*p* < 0.001). Moreover, the expression levels of *RERE-AS1* (*p* < 0.05), *TFAP2A-AS1* (*p* < 0.01), and *MIR302CHG* (*p* < 0.05) varied according to tumor stage (stage I, II, III, and IV), *COL4A2-AS1*, *RERE-AS1*, *NDUFA6-DT*, and *MIR302CHG* varied according to T stage (T1, T2, T3, and T4). However, in the subgroup analyses based on the N stage (N0, N1, N2, and N3), only *BAIAP2-DT* and *FARP1-AS1* were differentially expressed in different N stages (Figure 3B).

### 3.3 Validity of the m7G-LPS

**FIGURE 4:**
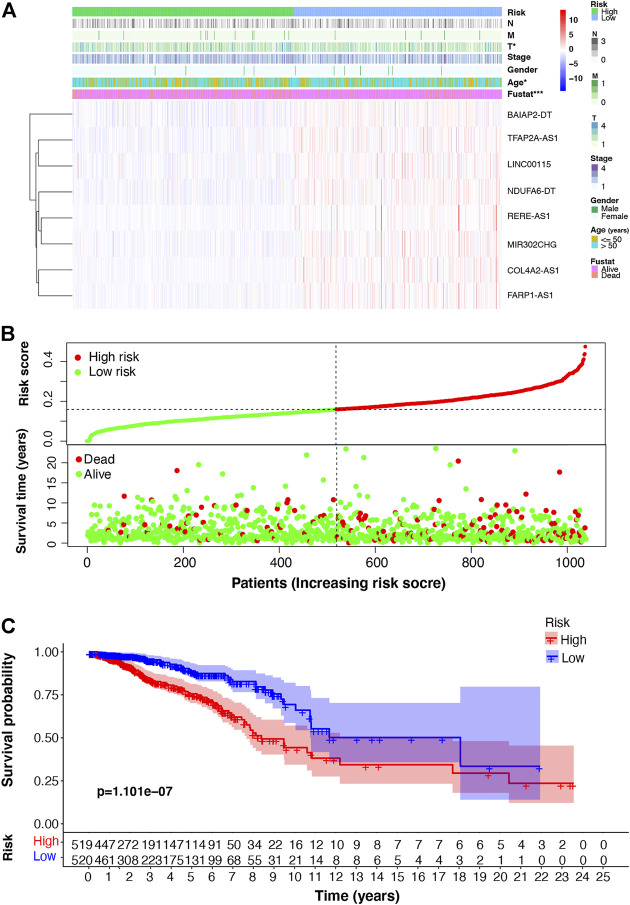
m7G-associated lncRNA (m7G-lncRNA) prognostic signature based on the eight prognosis-related m7G-lncRNAs. **(A)** Heat map of the difference in expression of the m7G-lncRNAs and clinicopathologic factors between the high- and low-risk groups **(B)** Distribution of the relationship between the risk score and patient survival status. **(C)** Kaplan–Meier survival analysis in the high- and low-risk groups (*p < 0.05; * * *p < 0.001).

Using the m7G-LPS, patients were classified into two subgroups based on whether the risk score was more than (high-risk) or less than (low-risk) the median of all patient risk scores. The heat map in Figure 4A showed that eight m7G-related lncRNAs are significantly differentially expressed between low- and high-risk groups. Besides, T stage, age, and survival status of patients with BC are related to risk subgroups. The risk curve and scatter plot showed an increased mortality rate with an increasing risk score (Figure 4B). Further, we investigated if m7G-LPS could predict survival by performing the survival analysis of Kaplan–Meier. The high-risk group exhibited remarkably worse OS compared to the low-risk group **(** *p* < 0.001; Figure 4C). These findings support that the m7G-LPS has prognostic value for patients with BC.

**FIGURE 5:**
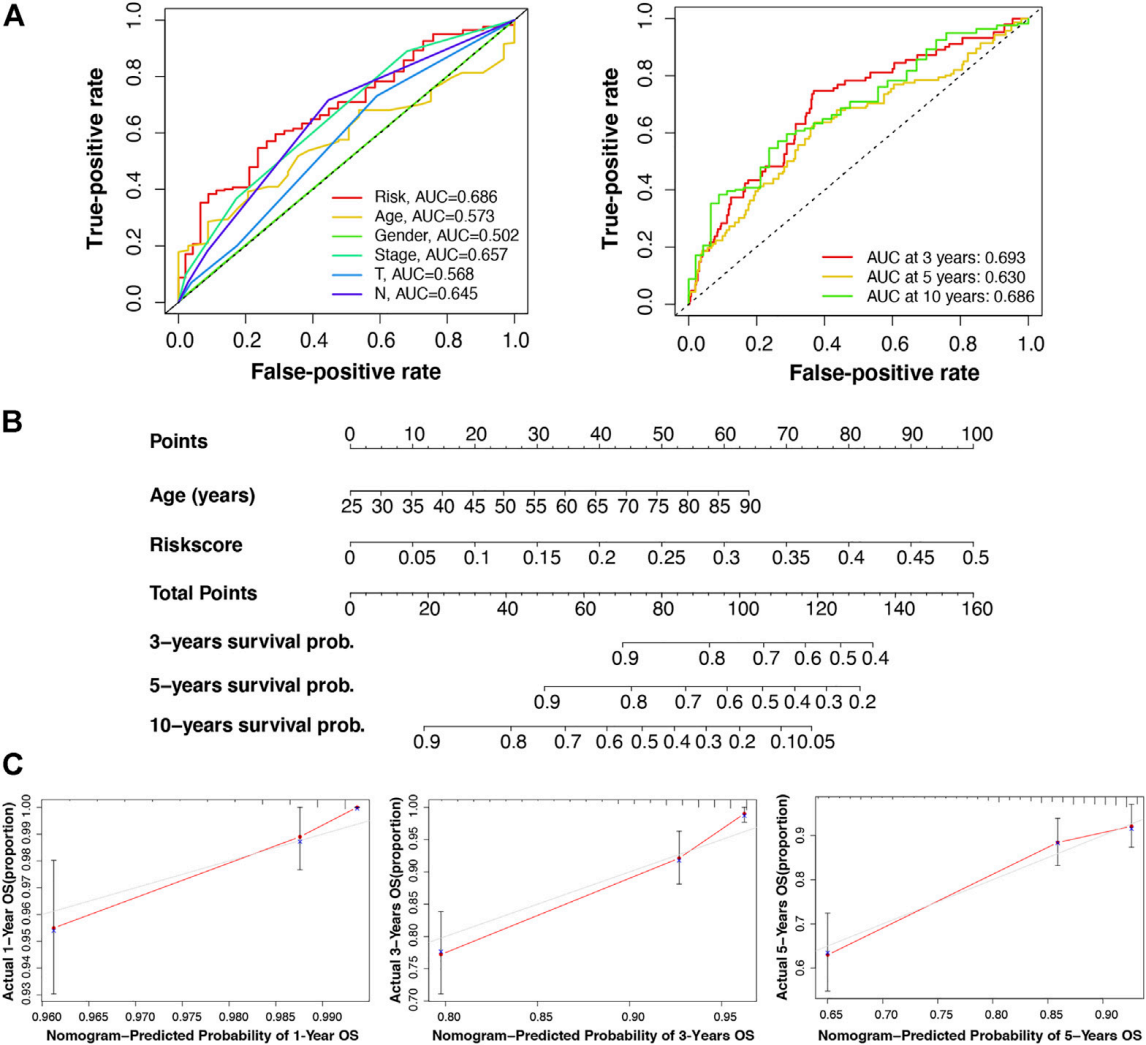
Validation of the reliability of the m7G-LPS and development of the nomogram. **(A)** Receiver operating characteristic curve of m7G-LPS and clinicopathological factors. **(B)** The nomogram developed based on the independent prognostic factors of age and risk score to predict the 3-, 5-, and 10-year survival rates. **(C)** The calibration plots of the nomogram were measured to evaluate the predicted probabilities of the nomogram.

Furthermore, we verified the prognostic value of the m7G-LPS and patient clinicopathological characteristics. The univariate and multivariate hazard ratio (HR) values of the risk score were 693 and 576, respectively, and all *p*-values were <0.001. This indicated that the risk score calculated through the m7G-LPS could serve as an independent predictor of prognosis in BC. In addition, univariate analysis showed that age, T, N, and M stages, but not gender, had significant prognostic value (*p* < 0.001). However, only age played an independent role in multivariate analysis (HR = 1.034, *p* < 0.001; Table 4). Based on ROC curve analyses, we found that the risk score yielded an area under the ROC curve (AUC) value of 0.686, which was the largest value among all clinicopathological factors. In addition, the 3-, 5-, and 10-year ROC curves showed corresponding AUC of the risk score of 0.693, 0.630, and 0.686, respectively (Figure 5A). These findings confirm that the signature can reliably predict the outcome of patients with BC. The prediction accuracy of the m7G-LPS was further validated. Thus, age and risk scores were included in the nomogram to better predict the 3-, 5-, and 10-year survival of patients with BC (Figure 5B). The calibration plots for the nomogram shown that the model calibration line is very close to the ideal calibration line, depicting good calibration (Figure 5C).

**TABLE 4:** Univariate and Multivariate analysis of m7G-LPS and clinicopathological factors.

Characteristics	Univariate analysis		Multivariate analysis	
	Hazard ratio (95% CI)	*p* value	Hazard ratio (95% CI)	*p* value
Age	1.033 (1.019–1.048)	<0.001	1.034 (1.019–1.049)	<0.001
Gender	0.866 (0.121–6.212)	0.886	0.555 (0.077–4.008)	0.559
Stage	2.149 (1.698–2.720)	<0.001	1.680 (0.999–2.826)	0.050
T	1.510 (1.216–1.876)	<0.001	0.945 (0.698–1.280)	0.715
M	6.481 (3.633–11.561)	<0.001	1.716 (0.750–3.926)	0.201
N	1.688 (1.401–2.035)	<0.001	1.165 (0.864–1.572)	0.316
Risk score	693.158 (71.684–6702.607)	<0.001	575.749 (57.656–5749.424)	<0.001

### 3.4 Differences in the m7G status of low- and high-risk groups and biological pathways of the m7G-LPS

**FIGURE 6:**
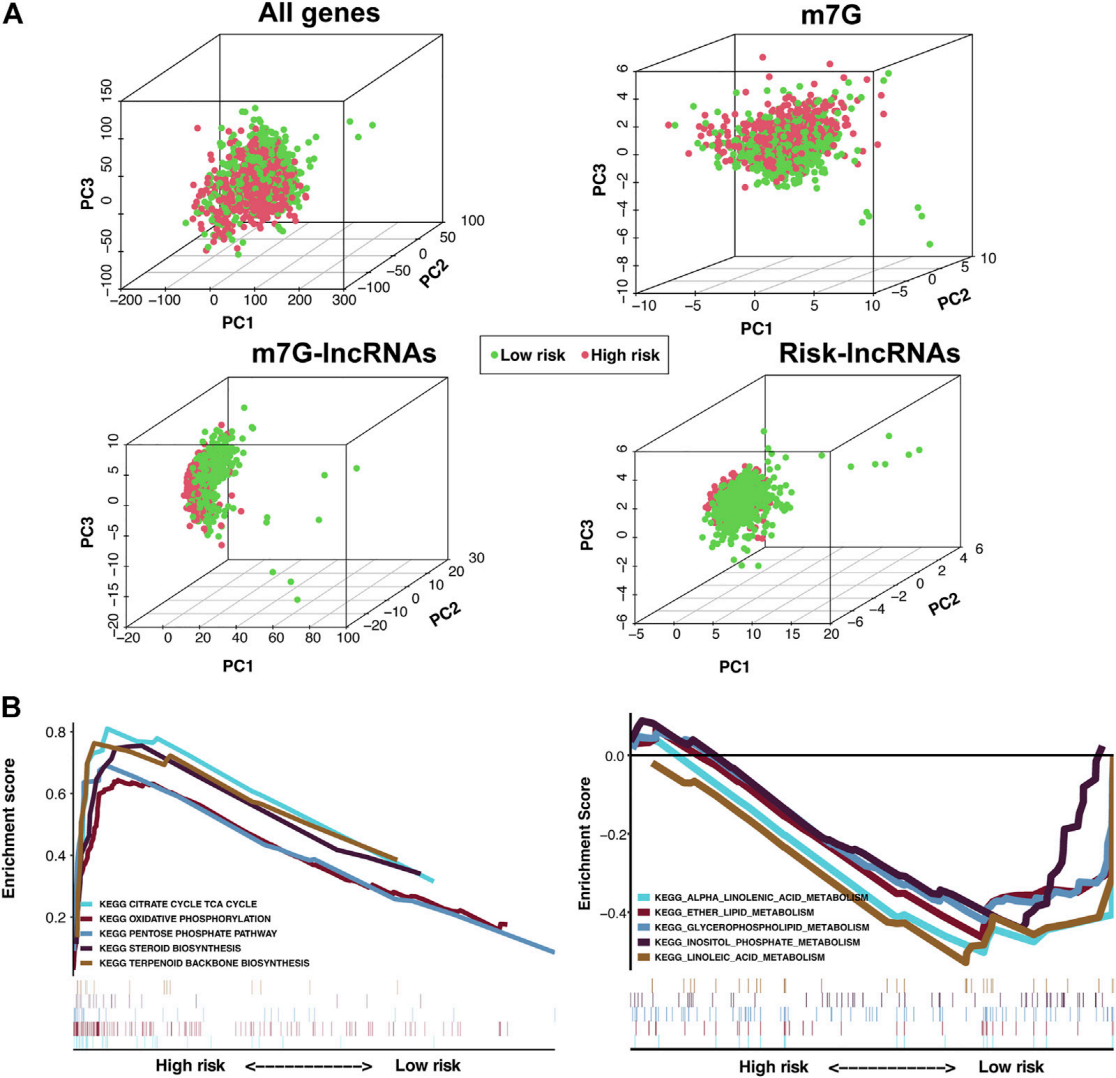
Principal component analysis (PCA) and GSEA analysis of the m7G-LPS. **(A)** PCA on the expression patterns of grouped samples based on the whole genome, m7G RNA modification-related genes, m7G-related lncRNAs, and the m7G-LPS expression profiles. **(B)** KEGG analysis of the m7G-LPS using GSEA.

PCA in four groups revealed that compared with the other three groups, BC samples in the m7G-LPS group could be better divided into two different groups (Figure 6A), further demonstrating the sensitivity and specificity of the m7G-LPS. To identify the potential biological signaling pathways underlying the molecular differences between high- and low-risk groups, we applied GSEA analysis. The result revealed that KEGG pathways, such as citrate cycle, TCA cycle, oxidative phosphorylation, pentose phosphate pathway, steroid biosynthesis, and terpenoid backbone biosynthesis, were remarkably enriched in high-risk samples, and alpha linolenic acid metabolism, ether lipid metabolism, glycerospholipid metabolism, inositol phosphate metabolism, and linoleic acid metabolism in low-risk samples (Figure 6B). The results indicated that m7G-related lncRNAs may involve in metabolism-related signaling pathway.

### 3.5 Correlation between m7G-LPS and tumor immune cell infiltration

**FIGURE 7:**
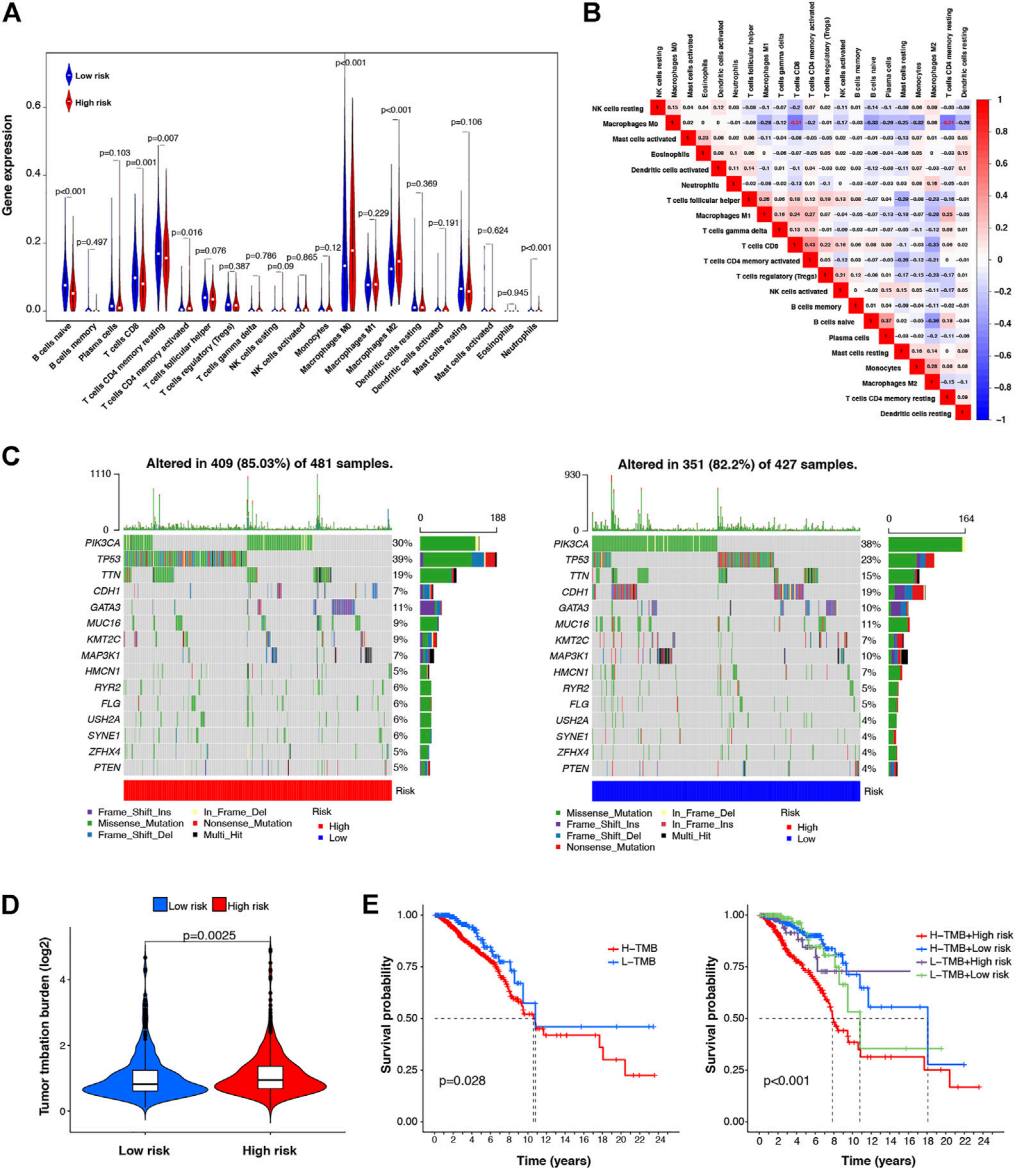
Correlation between the m7G-LPS and infiltrating level of immune cells and TMB. **(A)** Violin plots of the infiltrating level of 21 types of tumor-infiltrating immune cells between high- and low-risk groups. **(B)** Spearman correlation analysis of 21 types of tumor-infiltrating immune cells. **(C)** TMB analysis in low- and high-risk groups. **(D)** Difference analysis of TMB between low- and high-risk group **(E)** Kaplan–Meier curve analysis of OS is shown for patients classified according to the TMB and risk score.

Tumor immune cell infiltration is closely related to tumor occurrence, invasion, and metastasis. Therefore, further investigation was conducted to explore whether the m7G-LPS risk score correlates with the expression of 21 tumor-infiltrating immune cell types. As shown in Figure 7A, naive B cells (*p* < 0.001), CD8 T cells (*p* < 0.001), resting CD4 memory T cells (*p* = 0.007), activated CD4 memory T cells (*p* = 0.0016), M0 macrophages (*p* < 0.001), M2 macrophages (*p* < 0.001), and neutrophils (*p* < 0.001) showed significantly different levels of infiltration between the low- and high-risk groups. Correlations between tumor-infiltrating immune cells in BC tissues are shown in Figure 7B. Both resting CD4 T memory and CD8 T cells showed a moderately negative correlation with M0 macrophages (r = –0.51 and –0.51, respectively).

### 3.6 TMB analysis and correlation with m7G-LPS

TMB is associated with immunotherapy efficacy and is emerging as a potential biomarker. To examine the underlying value of TMB in BC, we performed TMB analysis through single nucleotide variation BC data in TCGA database to assess cancer-associated gene mutation frequency. Figure 7C shows a high TMB (85.03%) in the high-risk group. Gene mutations were more frequent in TP53 (39%). In the low-risk group, the proportion of samples with mutations was 82.2%, slightly lower than that in the high-risk group, and PIK3CA was the most frequently mutated gene (38%). In addition, our risk subgroup-based analysis, shown in Figure 7D, manifested that the TMB of high-risk patients was higher than that of low-risk patients. Subsequently, according to the TMB score, we classified all patients into low- and high-TMB groups. We further analyzed the value of the prognosis of TMB in patients with BC by Kaplan–Meier survival analysis. Our findings indicated worse OS for high-TMB patients by contrast with low-TMB patients. In combination with the prognostic signature, we found that patients with BC with both high-TMB and high-risk scores had the worst prognosis. The above data indicate that TMB has prognostic value in BC, and combined with the risk model, it can better predict the outcome of patients with BC (Figure 7E).

### 3.7 RT-qPCR validation of differentially expressed m7G-related lncRNAs

Univariate Cox regression and lasso Cox regression analysis was used to screen prognostic m7G-related lncRNAs, which were used to construct m7G-LPS. Further studies are required to validate the findings of lncRNAs, so we conducted the RT-qPCR test *in vitro*. Supplementary Figure S4 shows *BAIAP2-DT*, *FARP1-AS1*, *NDUFA6-DT*, *MIR302CHG*, *and TFAP2A-AS1* high expression in MCF-7, and *LINC00115* low expression in MCF-7.

## 4 Discussion

Worldwide, BC is the most common malignant tumor in women (Momenimovahed and Salehiniya, 2019). Despite continuing advancements in the multidisciplinary approach to its treatment (Waks and Winer, 2019), BC remains the primary killer of women with cancer. In recent years, with the development of bioinformatics and the application of high-throughput sequencing, m7G modification has been recognized as playing a key role in RNA splicing, stability, and efficient translation (Malbec et al., 2019; Chen K. et al., 2021, 0; Zhang et al., 2021). However, functional studies of the m7G modification-related lncRNAs remain limited. Therefore, in this study, we selected and validated eight differentially expressed m7G-related lncRNAs with prognostic values in BC, namely, *BAIAP2-DT*, *COL4A2-AS1*, *FARP1-AS1*, *RERE-AS1*, *NDUFA6-DT*, *TFAP2A-AS1*, *LINC00115*, and *MIR302CHG*, created m7G-LPS, and conducted a combined analysis of the clinicopathological characteristics, tumor immune cell infiltration, and TMB to examine the role of m7G-related lncRNAs in BC. We found that the m7G-LPS can predict the clinical outcome of patients with BC and evaluate the immune cell infiltration of tumors and TMB in BC. Model-lncRNAs can be used as diagnostic lncRNA biomarkers and may serve as potential therapeutic targets. Overall, the m7G-LPS discovered in this study extends the concept of post-transcriptional modifications of lncRNA, paving a path toward the exploitation of new measures for disease prevention, early detection, and therapy, ultimately contributing to improving patient prognoses.

Currently, many studies have constructed prognostic models of m7G-related lncRNAs by analyzing transcriptomic data from open databases, which can be used to estimate the prognosis of cancer patients (Song et al., 2021, 2022; Liu et al., 2022). For instance, in esophageal squamous cell carcinoma, a prognostic model constructed using seven prognostic m7G-related lncRNAs could predict the prognosis of patients, and the risk score calculated through risk signature was strongly associated with the level of immune cell infiltration (Zhao et al., 2022). In clear cell renal cell carcinoma, 12 prognostic m7G-related lncRNAs were screened, and the constructed model proved to have good accuracy and reliability in predicting OS (Ming and Wang, 2022). In hepatocellular carcinoma, m7G-LPS showed clinical value in predicting outcomes, immunotherapy effects, and drug sensitivity in patients with hepatocellular carcinoma (Wei et al., 2022). Collectively, these findings and those of previous studies support that m7G-related lncRNAs could serve as prognostic and diagnostic biomarkers for cancer, helping treatment selection and disease monitoring. In addition, these m7G-related lncRNAs could be therapeutic targets for BC. In this study, we created an m7G-LPS based on eight prognostic m7G-related lncRNAs and confirmed its prognostic value. The risk score, combined with age, is an independent prognostic factor for patients with BC, according to univariate and multivariate Cox regression analyses. Further, the AUC value of the ROC curve also showed that the prognostic signature has high prediction accuracy. Thus, for a more objective prediction of the 3-, 5-, and 10-year survival rates of patients with BC, we created a nomogram on the basis of age and the risk score. Results of PCA showed that compared with the whole-genome, m7G-related genes, and m7G-related lncRNAs, the expression profiles in the m7G-LPS group could better distinguish between low-risk and high-risk patients. Thus, the m7G-LPS has independent value and extremely high reliability and specificity for predicting BC prognosis. To the best of our knowledge, this is the first predictive signature of BC prognosis based on m7G-related lncRNAs and will likely be further refined by incorporating accumulating data.

m7G modification is indispensable for RNA metabolism, processing, and function. It is involved in tumor development, progression, and response to therapy. METTL1 methyltransferase mediates m7G methylation in Let-7e-5p miRNA and modulates the malignant phenotype of cell migration through its catalytic activity (Pandolfini et al., 2019). In addition, METTL1 also mediates Arg-TCT-4-1 tRNA modification, driving oncogenic transformation by remodeling the mRNA “translatome” (Orellana et al., 2021). In lung cancer, m7G methylase also regulated the m7G modification level of tRNA, promoting lung cancer growth and invasion. In this research, we also attempted to analyze the potential biological functions of the m7G-LPS using GSEA. The Kyoto Encyclopedia of Genes and Genomes pathway enrichment analysis showed that metabolism-related pathways were the most enriched in the high- and low-risk group. Diseases are often caused by deregulated metabolic signaling (Beckwith et al., 2018, 80), including BC (Akella et al., 2019). On the one hand, emerging evidence suggests that oncogenes and tumor suppressor genes in cancer, which usually include MYC, HIF, P53, and RAS, regulate the metabolic phenotype of tumor cells and inhibit the TCA cycle, diverting glutamine to fuel the TCA cycle (Anderson et al., 2018). In contrast, previous studies confirmed that the stability of the pentose phosphate pathway is crucial for the cell cycle, proliferation, and metastases (Lin et al., 2018; Leal et al., 2019). Thus, through enrichment analysis, we can surmise that the m7G-LPS may promote BC by regulating metabolic pathways.

Tumor-infiltrating immune cells, key components of the tumor microenvironment, are reportedly useful in predicting cancer prognosis, including that of BC (Bense et al., 2017). Thus, immune cells have emerged as a novel therapeutic target for cancer (Lazăr et al., 2018). For instance, CD8^+^ T cells are associated with tumor size, lymph node status, Ki-67 index, and molecular subtypes of BC. In addition, infiltration of CXCL13-expressing CD4^+^ follicular helper cells is predictive of BC prognosis (Zhu et al., 2016). B cells that are acutely activated may be involved in eliminating early tumor cells or tumor clearance through classical immunoglobulin-mediated mechanisms (DeNardo and Coussens, 2007). Here, we used TCGA data to investigate the correlation between the m7G-LPS and the tumor immune cell infiltration levels. We found significantly different levels of infiltrating naive B cells, CD8 T cells, resting CD4 memory T cells, activated CD4 memory T cells, M0 macrophages, M2 macrophages, and neutrophils between the low- and high-risk groups, with higher infiltration levels in the low-risk group, compared to the high-risk group. Our work indicates that the m7G-LPS have the ability to predict the infiltration levels of tumor immune cells in BC, thus, more accurately predicting the prognosis of BC.

The TMB conceptually represents the total number of mutations in a tumor sample. Tumor mutations cause the presence of immunogenic neoantigens on the surface of carcinoma cells. In general, the more mutations (i.e., the higher the TMB), the greater the likelihood that neoantigens presented by MHC proteins will be immunogenic, which aids T cells in recognizing and eliminating carcinoma cells (Rooney et al., 2015; Chabanon et al., 2016). However, 5% of patients with BC have high TMB, primarily patients with metastatic BC. A study has shown that TMB is a novel biomarker of immune checkpoint inhibitor sensitivity. Compared with the immunohistochemistry detection of PD-1 and PD-L1 expression, TMB is more effective in predicting the immunotherapy of patients with tumors, treated with PD-1 and PD-L1 inhibitors. Therefore, patients with high TMB may benefit from immune checkpoint inhibitors (Barroso-Sousa et al., 2020). In this work, we also preliminarily examined the correlation between M7G-LPS and TMB. We found that *TP53* gene mutation was mainly found in patients with a high-risk score, and TMB was higher in patients with low-risk scores. In the prognostic analysis, we found that patients with high TMB had poor prognoses. The survival outcome of patients with high TMB and high-risk scores was the worst in the entire cohort. The above results indicate that M7G-LPS is helpful in predicting the TMB of patients with BC, and the combination of TMB and prognostic m7G-related lncRNAs as biomarkers may help predict the patient outcome and guide the selection of immunological treatment.

There are limitations in our study. First, we constructed a m7G-LSP based on prognostic and differentially expressed m7G-related lncRNAs, however we have not yet found other data sets that included expression of eight lncRNAs, clinicopathological characteristics, and follow-up data. Thus, the m7G-LSP could not be verified further. In addition, we verified the expression levels of all eight m7G-related lncRNAs *in vitro*, but further functional experiments are needed in future. We leave further verifications as future work.

In conclusion, we identified a novel and reliable prognostic signature based on eight m7G-related lncRNAs. *BAIAP2-DT*, *COL4A2-AS1*, *FARP1-AS1*, *RERE-AS1*, *NDUFA6-DT*, *TFAP2A-AS1*, *LINC00115*, and *MIR302CHG* were screened as diagnostic biomarkers. Further improvement and validation to refine the predictive signature, nomogram, and diagnostic biomarkers might provide the necessary evidence for its adoption into clinical practice, drive the relentless improvement in prognostic information, and provide new prognostic biological targets for patients with BC.

## Data Availability

The original contributions presented in the study are included in the article/Supplementary Material, further inquiries can be directed to the corresponding author.
