# Supplementary material for: A novel prognostic signature based on N7-methylguanosine-related long non-coding RNAs in breast cancer
Source: Front Genet. 2022 Oct 13;13:1030275. doi: 10.3389/fgene.2022.1030275 (PMC9608183; doi:10.3389/fgene.2022.1030275)
Supplement: Supplementary file 1 [file DataSheet1.docx]

Supplementary Material


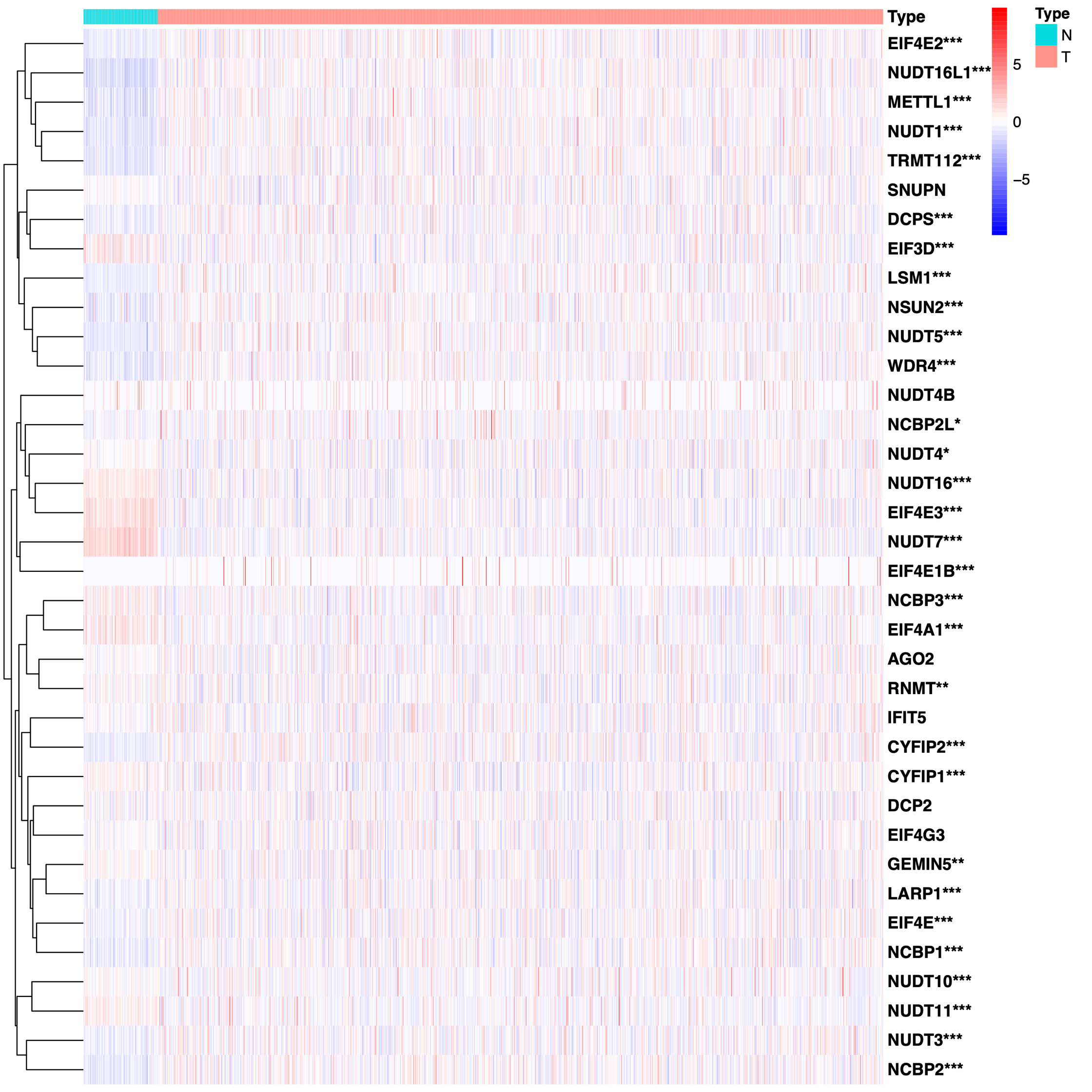


Supplementary Figure 1. The heat map of m7G-related gene differential expression analysis.


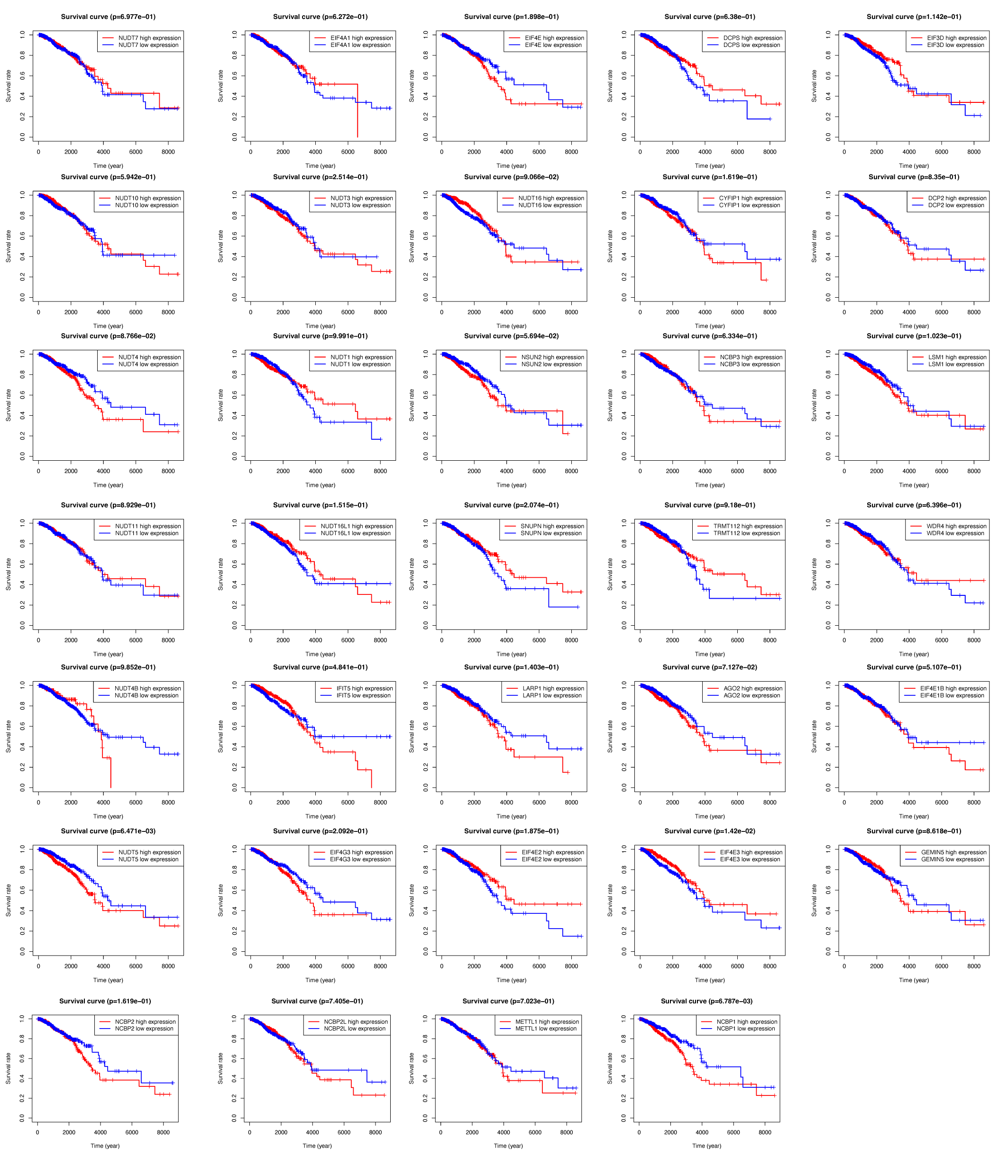


Supplementary Figure 2. The Kaplan–Meier survival analysis of m7G-related genes.


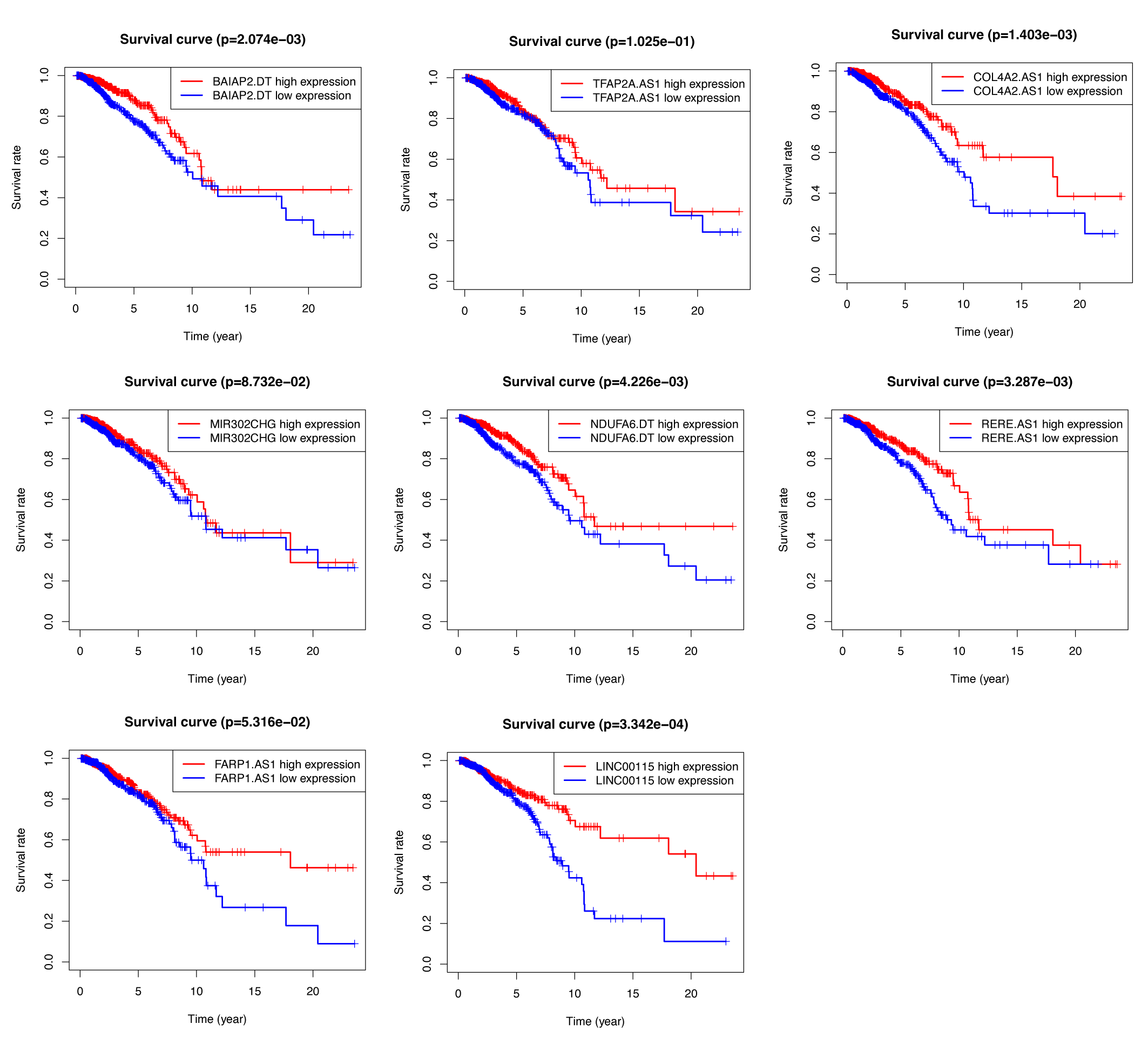


Supplementary Figure 3. The Kaplan–Meier survival analysis of eight m7G-related lncRNAs


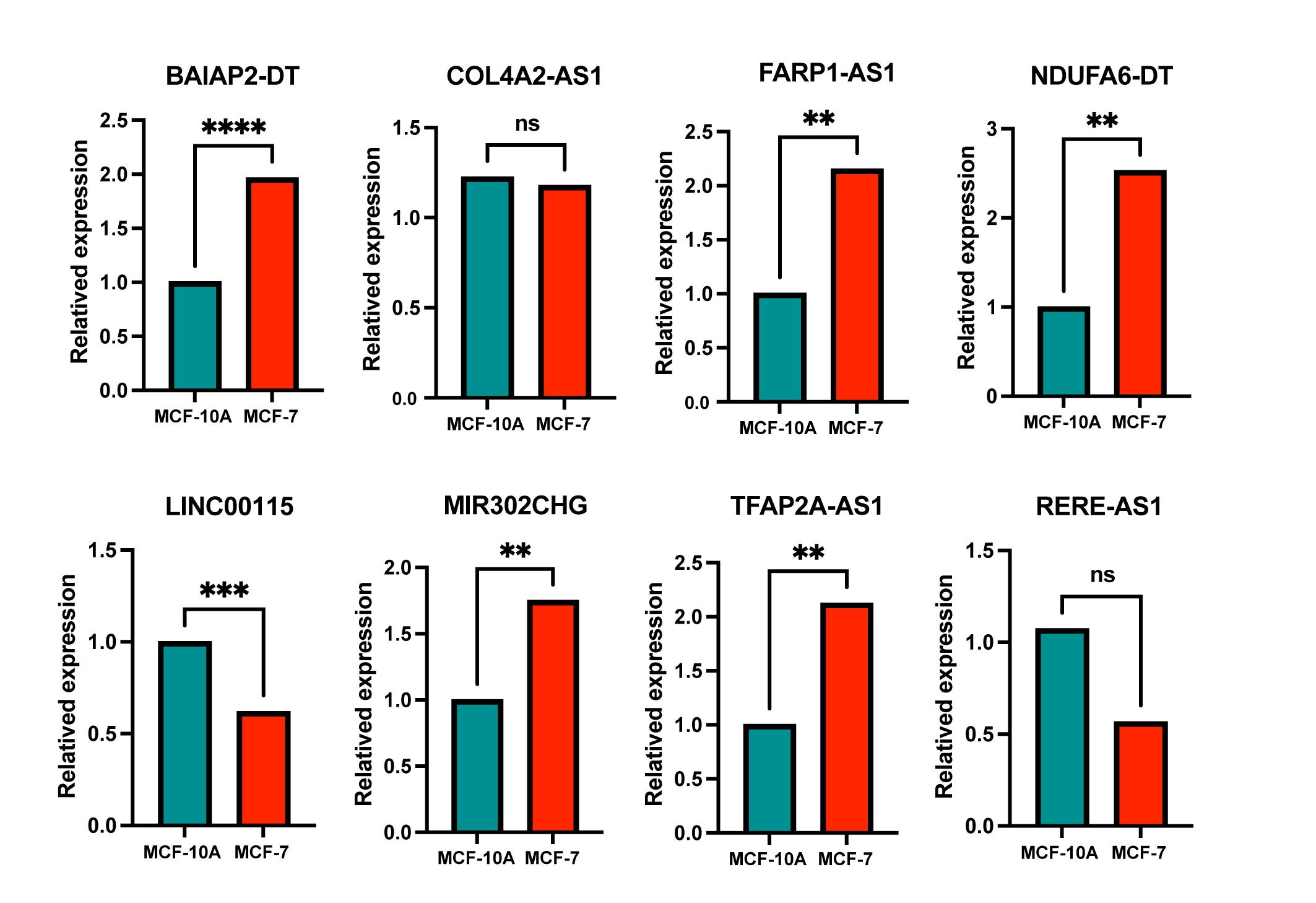


Supplementary Figure 4. RT-qPCR verified the expression levels of eight m7G-lncRNAs. (ns: not significant, *p < 0.5, **p < 0.01, ***p < 0.001, ****p < 0.0001)
